# Twelve Tips for COVID-19 friendly learning design in medical education

**DOI:** 10.15694/mep.2020.000103.1

**Published:** 2020-05-20

**Authors:** Jorge Reyna

**Affiliations:** 1 The Royal Australia and New Zealand College of Ophthalmologist - RANZCO

**Keywords:** learning design, online delivery, medical education, online learning, student-centred approach, technology-enhanced learning

## Abstract

This article was migrated. The article was marked as recommended.

The COVID-19 pandemic has caused educational institutions around the world to close their doors and move their teaching and learning into the online space. For many medical educators, who usually rely on face-to-face and blended instruction, this presents a challenge. By rethinking learning design, medical educators can ensure a smooth transition of their subjects into the online space. However, because of the immersive nature of medical education, not all teaching and learning activities can be delivered online. This paper outlines twelve tips using evidence-based educational practices and a student-centred approach. The twelve tips presented and discussed in this paper can help medical educators to transition to online learning and maintain the integrity of their subjects. They can also promote student self-regulation, help develop graduate attributes, and generally enhance learning experiences during pandemic social distancing. Finally, these tips can be used to rethink medical education in the post-pandemic era.

## Introduction

The COVID-19 pandemic has pushed the world into quarantine and moved work, learning, socialising, and leisure to the online space. Digital technology availability has made it possible to rethink all these activities. In the higher education landscape, universities have been delivering blended and online learning for more than two decades ( Bonk & Graham, 2012), but the pandemic has forced medical schools to close and created a challenge for medical educators across the world. Due to the immersive nature of medical education ( Patel, 2016), where wet labs, tutorials, and internships are essential, this has impacted how students learn. Demonstrators now run these sessions virtually, mediated by digital technologies such as web conferencing applications ( Gegenfurtner & Ebner, 2019). Many institutions have reduced the overall hours of casual tutoring, increasing the pressure and the challenge of delivering compelling learning experiences. The current COVID-19 situation is creating new opportunities for medical educators to trial innovative learning designs to provide the best possible learning experiences for students.

Evidence-based education has been implemented extensively in medical education in the last twenty years. There is a wide range of theoretical models ( Gordon *et al.,* 2019), learning theories ( Mukhalalati & Taylor, 2019), pedagogies ( Pasarica, Kay & Cameron, 2019), and instructional strategies ( Fatima, Naz, Zafar, Fatima & Khan, 2020) which could improve the student learning experience. In conjunction with digital technologies, these can provide new opportunities to make medical education more relevant to the times we live in ( Bates, 2016). The field of learning design has been growing in the last decade with the support of digital technologies ( Dalziel, 2015). Educational concepts such as constructive alignment ( Biggs and Tang, 2011), outcome-based education ( Sideris *et al*., 2020), active learning strategies ( Reyna, 2015a), authentic assessments providing students with meaningful feedback ( Tepper, Bishop & Forrest, 2020), students as partners ( Reeves, Kiteley, Spall & Flint, 2019), mobile learning ( Lall *et al.*, 2019), and transition pedagogies ( Atwa, O’Connor & Hegazi, 2019) are some examples of an evidence-based approach to medical education.

The medical curriculum now has a student-centred approach integrated with clinical and scientific principles ( Johnson, Owens & O’Neil, 2019), competency-based education ( Crawford, Cofie, McEwen, Dagnone & Taylor, 2020), self-directed learning ( Caramori *et al.*, 2019), team-based approaches ( Graves, 2019), and personalised learning ( Schwinn, Cooper & Robillard, 2019). Medical education aims to produce well-rounded new practitioners who demonstrate problem-solving and communication skills, engagement with the needs of society, an understanding of cultural diversity, and reflective skills. Applying evidence-based educational principles to medical education can promote a more humanising and critical educational practice. COVID-19 is providing a new opportunity to enhance student learning experiences.

This article provides the medical education community with evidence-based practices to redesign learning, taking advantage of digital technologies widely available to educators. The twelve tips are organised in a logical progression from a learning design perspective and take a student-centred approach, from engaging students as partners in the learning process to creating effective teacher presence, using online modules, visual design and aesthetics, flipped learning, webinar delivery, rethinking assessment and feedback, and modelling student self-regulation.

## 1. Engaging students as partners in learning design

There is a new tendency in higher education to empower students as partners in the learning process ( Matthews *et al.,* 2019). This approach enables them to be part of the learning design and promotes engagement, motivation, and agency ( Becker, 2019). Research has shown that the strategy of making students partners increases their confidence and self-efficacy ( Gravett, Kinchin & Winstone, 2019). Involving medical students as partners will increase educators’ understanding of how new generations learn and could potentially enhance student-educator relationships. Like ‘mutualism’ in biology ( Boucher, 1985), the strategy can be mutually beneficial to both parties. Another advantage of this approach is that it could increase student awareness of metacognitive learning, self-evaluation, and reflection and increase their sense of belonging to the institution or discipline ( Cook-Sather, Bovill & Felten, 2014). Possibly mediated by the protagonist role of students and its effect on self-regulation and motivation, the strategy of students as partners can enhance the learning experience.

Inviting students to a focus group at the end of the semester to discuss troublesome learning issues could achieve this kind of partnership. By identifying what students are struggling within their studies, this will provide an opportunity to restructure learning tasks to enhance learning experiences. For instance, knowing the difficulties students encounter could lead to the redesign of a lecture into a webinar which ‘flips’ the troublesome content. This way students can engage with the content as many times as required before coming to the webinar, ready to engage in higher-order thinking via discussion and consideration of ideas. Other strategies for students as partners include letting them suggest how to improve a tutorial session, working with them to develop marking rubrics, and having them create or curate content for the subject.

## 2. Use video vignettes to create online teacher presence

The multimedia principle suggests that students learn better when the learning materials contain words and images ( Mayer, 2008). The ‘personalisation principle’ means engaging the learner by delivering content in a conversational tone to enhance learning (Kurt, 2011). Through the design of online subjects, facilitation of discourse, and direct instruction, educators can establish an online teaching presence ( Orcutt & Dringus, 2017). A robust online teaching presence makes for a robust online learning experience and a sense of community for students ( Bates, 2016).

A video is an excellent tool to create online teacher presence with medical students. By creating weekly introductory videos on the subject being taught, educators can create a strong teacher presence. The videos should be one or two minutes maximum to engage students effectively. Video scripting is essential to avoid redundant words ( Stockman, 2011). One minute of video is about 130-150 words, so scripting is essential to avoid overly long videos ( Carroll, 2014). In the videos, educators should talk about what the learning outcomes of the week will be and how students will learn with the activities proposed. It is essential to use a tripod and to position the face following the ‘rule of thirds’ ( Paulo, Casteleira & Leão, 2008). Dividing the screen evenly into thirds, both horizontally and vertically, and positioning the talking head at the intersection of those dividing lines, creates an effective composition. High-quality advertising in social media like Instagram or Facebook has exposed most students to high-quality visual content, and so they will have high expectations of their learning materials. Applying these recommendations will make the content credible and engaging for students.

## 3. Deliver lectures as interactive modules

Converting face-to-face lectures into online delivery is not about just recreating and recording them to put them onto the Learning Management System (LMS). That approach can be used effectively for lecture revision, but it is problematic as a primary source for online learning ( Reyna, 2015b). The way people learn face-to-face is substantially different in online environments. People have a short attention span when looking at a monotonous screen, especially a talking head with no interaction ( Snelson, 2011). Converting traditional lectures into interactive modules is time-consuming but worthwhile because it will enhance the learning experience.

The first step is putting PowerPoint content into a Word document and using the ‘segmentation principle’ from multimedia learning ( Mayer, 2008) to guide division into sections. In other words, dividing content into segments to improve retention. Then, use a learning design sequence in each section that presents content, worked examples, activities, and feedback. Present the content using a combination of images, text, and voice-over. A worked example could be a case scenario based on text and image or video. The activity could be a multiple-choice question with automatic feedback. Educators should use open learning resources where available, rather than creating them, which can be a time-consuming process. Technology to deploy the learning design could be e-learning authoring tools such as Captivate, Articulate Storyline, iSpring, or Camtasia Studio ( Giacumo & Conley, 2015). If the institution does not have licences for such software, the H5P platform offers a free-of-charge online application to create learning modules ( Rekhari & Sinnayah, 2018). When designing a module in H5P, it is possible to share it via a link or embed it into the LMS. The disadvantage is that tracking of student completions is not possible unless the institution can integrate H5P with its LMS.

## 4. Use visual design and aesthetics to improve online delivery

Visual design and aesthetics have a profound effect on how students engage with online learning materials ( Malamed, 2015). Regrettably, these principles (layout design, colour theory, typography, use of images, and video principles) are not well-known in medical education ( Reyna, Hanham, & Meier, 2018). The evidence behind these principles comes from multiple disciplines, including neuroscience ( LeDoux, 1989), psychology ( Chang, Dooley, & Tuovinen, 2002), multimedia learning ( Mayer, 2008), and visual design ( Lin, 2013). Disciplines like marketing and product development use these principles to communicate messages promoting goods and services. Using these principles in online learning is important for credibility and helps students focus on content rather than on figuring out navigation. Applying these principles could improve cognitive engagement with content and promote usability and accessibility. There is a new initiative in this field, ‘universal design’, that aims to be inclusive and to enhance the learning experience for everyone, not only students with disabilities ( Kerr, McAlpine & Grant, 2014).

Layout design is the placement of the design elements (images, text, videos) in an interface (e.g. PowerPoint, online module, website, poster), helping the student to navigate the content and promoting cognitive engagement ( Reyna, 2019b). A symmetrical layout places text and images in an order which creates balance and is easy on the eye. Colour is also essential, but educators need to be careful about how they use it in the online space. For instance, using red and green in graphics will disadvantage students with a colour deficiency ( Bader & Lowenthal, 2018). Typography affects legibility and may cause problems for students with dyslexia. For instance, brush-script fonts are hard to read, but sans-serif fonts such as Arial, Verdana, or Helvetica will make the text more legible ( Malamed, 2015). The use of images should have a purpose, such as reinforcing the message or making a complex scientific process easy to understand, and they will serve as visual cues for students to remember. When recording short video vignettes, be sure to apply the ‘rule of thirds’, use a tripod to keep the picture stable, and script them for maximum impact ( Stockman, 2011).

## 5. Use flipped learning to develop lifelong learning skills

The fact that COVID-19 has forced a move to purely online delivery does not mean educators can’t flip learning activities anymore. The literature describes many advantages of flipped learning in medical ( Moffett, 2015) and health science disciplines ( Hew & Lo, 2018). By asking students to prepare before their online lecture, they are similarly becoming responsible for their own learning and developing lifelong learning skills ( Abeysekera & Dawson, 2015). As medical professionals, students will need to engage with the latest evidence-based interventions to improve patient outcomes ( Sharma, Lau, Doherty & Harbutt, 2015). Modelling lifelong learning skills for medical students is a highly desirable aspect of flipped learning. A good flipped learning strategy considers seven elements: pedagogies; instructional strategies; lesson plans; what happens before, during, and after class; flipped learning enablers such as the visual design principles discussed above; building and testing the necessary technology; and communicating to students their need to prepare for the classroom ( Reyna, Davila, & Meier, 2016).

Several models can inform flipped learning interventions, most of them considering what happens before, during, and after the classroom ( Mazur, Brown & Jacobsen, 2015; Moffett, 2015). Learning outcomes should aim for higher-order thinking when possible, rather than simply understanding and remembering. There is a novel model available that considers self-regulation and motivation as the drivers of successful flipped learning experiences ( Reyna, 2019a). If students do not engage in preparation before the online lecture, the intervention can fail. Self-regulation skills such as goal setting, environment structuring, time management, task strategies, and self-consequences come in handy ( Barnard-Brak, Paton & Lan, 2010). These skills can be benchmarked at the beginning of the semester via a simple, validated, flipped learning survey ( Kenney & Newcombe, 2018). Understanding students’ self-regulation skills before a flipped learning intervention could help educators to identify student needs regarding study skills. Students can develop or improve their self-regulation skills with appropriate training ( Zimmerman, 2002).

## 6. Use webinars to deliver tutorials

Using webinars is one of the best ways to deliver engaging and interactive online lectures and tutorials. To ensure webinars are useful, educators need to formulate clear learning outcomes for sessions, using higher-order thinking verbs, and align them with learning activities and assessments ( Biggs & Collis, 1982). Pedagogical considerations include active learning pedagogies such as Problem-Based Learning ( Caramori *et al.*, 2019), Inquiry-Based Learning ( Capaldi, 2015), Collaborative Learning ( Magen-Nagar & Shonfeld, 2018), case studies ( Herreid & Schiller, 2013), and Peer Learning ( Topping & Ehly, 1998). Using breakout rooms, students can be assigned randomly to a group and work with their peers for 10-15 minutes discussing a topic, afterwards returning to the main room and summarising their discussions ( Knipfer *et al.,* 2019). As a facilitator, it is essential to deliver a session that encourages students to participate by using polls, quizzes, and questions to promote critical thinking ( Bates, 2016). With flipped learning, educators can assess student understanding of preparatory material and address possible misconceptions or reinforce learning in real-time ( Moffett, 2015). A desirable strategy is to present and discuss complex concepts at the beginning when students are more likely to be focused. A lesson plan and following it carefully are essential to ensure the tutorial will cover the learning objectives.

As an educator, it is essential to find ways to keep energy levels up while presenting and to enthusiastically demonstrate to the students why the topic is vital ( Sharan & Carucci, 2014). Some strategies include changing voice pitch and speed of delivery to engage students. Ensure a break every ten minutes with an activity or questions to avoid monotony. If presenting slides, educators should use a minimalistic slide design with short sentences and diagrams or pictures. Font size should be 40pt for titles and 20-24pt for a paragraph ( Carter, 2012), using the visual design and aesthetic principles discussed above ( Malamed, 2015).

## 7. Rethink assessment tasks

Medical students need to develop a set of graduate attributes, also called ‘soft skills’, which need to be embedded in curricula. These skills include communication skills (Park, Kamin, Son, Kim & Yudkowsky, 2019), cultural awareness ( Malau-Aduli, Ross & Adu, 2019), reflective practice ( Wald, White, Reis, Esquibel & Anthony, 2019), groupwork and collaboration ( Parker, Hodierne, Anderson, Davies & Elloy, 2019), and conflict resolution skills ( Roth, Eldin, Padmanabhan & Friedman, 2019). The key to success for medical professionals is thus the promotion of authentic assessments where students demonstrate understanding and application of the relevant science (anatomy, physiology, pharmacology, or pathology) as well as a wide range of graduate attributes.

Authentic assessments in medical education include groupwork activities like online presentations, digital media projects, writing scientific journals and mini-reviews, writing grant applications, panel discussions and mini-conferences using webinar platforms, doctor-patient roleplay, reflective tasks, and virtual consultations. These strategic assessments are appropriate in the first and second year to develop the graduate attributes required. Students can work entirely online with their peers by using platforms such as Google Drive, Microsoft Teams, WhatsApp, and social networks. Educators should consider how technology can enhance teaching and learning. By using models such as SAMR (Substitution, Augmentation, Modification or Redefinition) ( Romrell, Kidder & Wood, 2014), a traditional assessment task can be a transformative learning experience for students. New technologies such as virtual, augmented, and mixed reality offer excellent ways to enhance learning experiences in medical education ( Bin & Zary, 2019).

## 8. Develop mechanisms to ensure healthy groupwork

Changing assessment tasks to online groupwork and collaboration will be ideal during the COVID-19 pandemic, but it brings challenges. As reported in the literature, students do not enjoy groupwork for various reasons ( Yudkowsky, Park & Downing, 2019). Groupwork can involve conflict between members or members not contributing to tasks or waiting for the last minute to act ( Poort, Jansen & Hofman, 2019). Other groupwork issues can include dominant personalities, introversion, personality clashes, lack of clear group goals, lack of task strategies, time management issues, lack of understanding of diversity, and intolerance towards others ( Davies, 2009). Recent research in science education has found that students enjoy working in groups when producing digital media assignments ( Reyna, Hanham, Vlachopoulos, & Meier, 2019). As everyone has different capabilities, group members feel the need to support their groups with the skills they have. Using digital media assignments with first and second-year medical students can model groupwork and collaboration to them, but it is also essential to teach students how to work effectively in groups.

There are excellent applications to ensure groupwork is effective, for instance SPARKPlus, a peer-review system for student contributions. Students start by rating themselves against a set of criteria or a marking rubric developed by the educator and then they move to rating their peers and leaving feedback ( Willey & Gardner, 2010). The criteria can be simple, e.g. (i) subject input for the project, (ii) punctuality and time commitment, (iii) contribution of original ideas, (iv) communication skills and working effectively as part of the team, and (v) focus on the task and what needs to be done. The system calculates a factor called Relative Performance Factor (RPF) that measures group contribution (0 to 1). The final mark for an individual student is the group mark multiplied by the RPF, so students who do not contribute effectively are penalised. If SPARKPlus is not available, a Google Form can also record data on student contribution. It will require calculations, but it will be as effective as SPARKPlus. It is crucial to articulate to students that their contributions to their groups will define their final grade.

## 9. Provide timely feedback to students

Timely feedback is essential, whether it is face-to-face, blended, or fully online. The purpose of feedback is to reduce discrepancies between understanding, performance, and the desired outcome ( Hattie & Timperley, 2007). Effective feedback works at four levels - task, process, self-regulation, and self-reflection. The task is about how the student understands the learning task, while the process is what is needed to complete it. Self-regulation is about how students self-monitor, directing and adjusting their actions to complete the task. Finally, self-reflection is about student reflections on the task ( Ramani, Könings, Ginsburg & van der Vleuten, 2019). Students need to be able to apply feedback to the next iteration of their assessment task. Ideally, assessment tasks should be built up within the subject. For instance, starting with a literature review early on the session and moving towards an experiment, then presenting the findings in a mini conference. Whether feedback is formative or summative, it requires structure.

Automatic feedback is highly desirable for online learning and quizzes on the LMS, or when producing interactive online modules. Verbal feedback can also be given during webinars. Audio feedback is one of the most efficient ways to provide feedback online to students ( Orlando, 2016). From the student perspective, audio feedback enhances the retention of content, the perception of instructor care, and the ability to understand nuance ( Ice, Curtis, Phillips & Wells, 2007). As a result, students tend to consider feedback more deeply and are more motivated to learn ( Rawle, Thuna, Zhao & Kaler, 2018). From the educator perspective, using audio feedback can deliver more feedback in less time. For instance, a study found that the mean times for text and audio feedback were 13.43 and 3.81 minutes, providing feedback of 129.75 words and 331.39 words, respectively ( Lunt & Curran, 2010). Educators can deliver audio feedback when marking assignments in Turnitin or with a free online application called Vocaroo ( Urgilés Echeverría, 2017).

## 10. Design open-book and take-home exams

Exams are an old fashioned and unnatural way to assess student learning ( Tapp & Martin, 2014). Sitting in exam conditions creates unnecessary stress for students and may not be the best way to gauge student preparedness for medical practice ( Cohen, Marshall, Cheng, Agarwal & Wei, 2000). In real life, medical and health professionals don’t face exams, but rather patients they need to help. It is understood that medical and health-related professionals need to demonstrate competencies for accreditation purposes to ensure patient safety. Even with strict accreditation, medical errors occur as the process of diagnosis and treatment, can be complicated ( Rodziewicz & Hipskind, 2019). Scientific concepts can be complicated, requiring students to ‘write’ them into long-term memory, also called schema ( Leppink & Hanham, 2019). Memorising concepts without any active learning component, such as medical internships, risks them being written into short-term or working memory, which is limited, and then vanishing a few weeks after the exam ( Baddeley & Hitch, 2017).

Open-book exams could potentially counter rote learning and promote medical students engaging in higher-order thinking and reflection. In formal settings, open-book exams can be restricted (e.g. students can bring their notes) or fully open (e.g. students can bring anything they wish). Open-book exam questions require skills and time to formulate items to ensure students cannot easily find the answer in a book. For instance, case-based scenarios with sophisticated multiple-choice alternatives to promote higher-order thinking are feasible ( Coughlin & Featherstone, 2017). Students need to already know and understand the foundational knowledge to be able to think critically and answer questions. During the COVID-19 period, the best approach will be take-home exams, but to invigilate these exams is almost impossible. Some companies provide online exam invigilation services, but the question to ask here is, shouldn’t we model academic integrity, social responsibility, and understanding the needs of patients to students? If a student is on board with these ethical concepts, it is likely they will exhibit fair-minded behaviour. Perhaps artificial intelligence will shortly make online invigilation services redundant because it will be possible to monitor students, and whether they are following exam procedures or not, anywhere they are.

## 11. Prepare and support students for online learning

Adjusting from blended to online learning is not as simple as it may sound. Educators will need the support of academic developers, learning designers, educational designers, instructional designers, and IT professionals. Also, students will need assistance, mainly because online environments require well-developed motivation and self-regulation skills. Self-regulation approaches to learning include students setting clear goals for their subjects, developing time management strategies, structuring their environment for productivity, designing task strategies, and accepting the self-consequences of their actions ( Barnard-Brak *et al.,* 2010). Educators can model these self-regulation subscales. Educators can curate online resources, such as YouTube videos, evidence-based blogs, and journal papers, to encourage medical students to become self-regulated learners.

Motivation is the sine qua non of self-regulation and is possible to model to students ( Zimmerman, 2002). Educators can help students to improve their self-efficacy with learning technology by providing support materials ( Agustiani, Cahyad & Musa, 2016). For instance, in teaching first-year medical students how to cite journal papers, educators could offer a basic tutorial developed with screencast software (e.g. Camtasia Studio) that guides them step-by-step in performing the task. To increase the ‘perceived task value’ of learning activities, educators need to ensure that the total marks allocated to a task are fair ( Vanslambrouck, Zhu, Lombaerts, Philipsen & Tondeur, 2018). For instance, asking students to write a literature review worth only 10% of their course grade will assign it a low task value, and students will have less motivation to complete it. ‘Attribution to failure’ - e.g. students blaming educators, the system, technology - is linked to poor performance ( Weiner, 2005), so it is essential to explain this to students and develop a reflective task to ensure they are on board. Finally, anxiety harms self-regulation ( Bembenutty, McKeachie, Karabenick & Lin, 1998). As educators, we can avoid causing students to feel anxious by writing clear subject learning objectives and accurate assessment instructions, communicating appropriately, using positive reinforcement, and offering them support.

## 12. Evaluate the learning design intervention

Every new learning design requires evaluation and feedback to improve the intervention. It is essential to have a holistic approach to student learning experience evaluation. Institutional student evaluation surveys sometimes don’t provide useable data to inform improvement. They tend to be too generic, especially when educators implement innovative learning design. Mixed-methods research, with data from student engagement with learning resources (LMS logs), course performance, open-ended questions, reflection, interviews, and focus groups with students and tutors, can evaluate the intervention and help to adjust the next iteration accordingly ( Phillips, McNaught & Kennedy, 2012).

It is essential before seeking evaluations of learning design by students to prompt them to reflect on their learning experience. Without doing this, students tend not to see the value of it. Using a reflective exercise, via reflective journals, message forums, or even webinar conversations, could promote a realistic student view of which learning activities worked and which ones require further refinement.

## Conclusion

Best practices to develop compelling online learning experiences for medical students should have a student-centred approach. This paper has highlighted the latest evidence-based principles which can engage medical students in meaningful learning during the current COVID-19 pandemic. Embedding evidence-based educational principles into the teaching and learning of medical professionals, in conjunction with the development of graduate attributes, will have a positive impact on patient outcomes. The twelve tips presented and discussed in this paper can be used by medical educators not only to enhance online learning, but also to improve teaching practices. A student-centred approach to medical education will help to prepare the medical educators of the future.

## Take Home Messages

• Best practices to develop compelling online learning experiences for medical students should have a student-centred approach.
• Evidence-based educational principles can engage medical students in meaningful learning during the current COVID-19 pandemic.
• Aligning learning design with subject learning objectives and graduate attributes will have a positive impact on medical students and patient outcomes.
• Principles covered in this paper will help to inform an evidence-based learning design during the COVID-19 era but also could help to rethink medical education.

## Notes On Contributors

Jorge Reyna is a senior learning designer with a vast experience in implementing evidence-based educational interventions in science education. Expertise in blended, online learning and using digital media to learn complex scientific concepts. His area of research includes self-regulation, motivation, group work dynamics and science communication. ORCiD: https://orcid.org/0000-0002-9909-0581
