# Supplementary material for: Twelve Tips for COVID-19 friendly learning design in medical education
Source: MedEdPublish (2016). 2020 May 20;9:103. [Version 1] doi: 10.15694/mep.2020.000103.1 (PMC10712607; doi:10.15694/mep.2020.000103.1)
Supplement: Peer review history

# Reviewer report: Reviewer response for version 1

**DOI:** 10.21956/mep.20071.r30838

**Published:** 2021-06-15

**Authors:** Suzanne Lady

**Affiliations:** 1 University of Western States

This review has been migrated. The reviewer awarded 4 stars out of 5 I enjoyed reading these 12 tips on teaching during Covid-19. I think the lessons we have learned during this pandemic will shape the future of medical education. Your paper beautifully points out strategies we should use regardless of whether we are teaching remotely or in a face-to-face environment. Keeping the strategies that worked well remotely will be an important part of continuous improvement. I especially enjoyed tip number seven: Rethink assessment tasks. It is not always possible to assess certain skills or knowledge remotely, but communication skills are perfect for online assessment. It is good for all of us to remember these tips as we make our way back into the classroom.

Reviewer Expertise:

NA

No decision status is available

# Reviewer report: Reviewer response for version 1

**DOI:** 10.21956/mep.20071.r30841

**Published:** 2020-05-24

**Authors:** Nandalal Gunaratne

**Affiliations:** 1 Faculty of Medicine, Wayamba University of Sri Lanka

This review has been migrated. The reviewer awarded 5 stars out of 5 This article is a godsend. It is practical and we'll written. There are many references.I learnt to use short screen casts, voice recordings, whatsapp chats, zoom discussions and the active learning modules in Moodle in the last two months. My students love it and are learning more enthsisatically.This article has given me more ideas and paved many paths. The references are going to be very useful too I am sure.Thank you and we'll done.

Reviewer Expertise:

NA

No decision status is available

# Reviewer report: Reviewer response for version 1

**DOI:** 10.21956/mep.20071.r30840

**Published:** 2020-05-21

**Authors:** Maria de los Angeles Fernandez-Altuna

**Affiliations:** 1 Universidad Nacional Autonoma de Mexico Facultad de Medicina

This review has been migrated. The reviewer awarded 4 stars out of 5 I like the approach of this article, its very practical specially for Med Schools. The twelve tips are logically organized so the reader can transit from engaging students, visual designg and estethics, to modules, assessment, feedback and student self regulation.This article has a comprehensive literature review and provides an excellent overview that could be helpful to many educators, specially during this COVID-19 pandemic forcing all schools to migrate to online teaching.

Reviewer Expertise:

NA

No decision status is available

# Reviewer report: Reviewer response for version 1

**DOI:** 10.21956/mep.20071.r30842

**Published:** 2020-05-21

**Authors:** P Ravi Shankar

**Affiliations:** 1 American International Medical University

This review has been migrated. The reviewer awarded 5 stars out of 5 I enjoyed reading this important paper on online teaching-learning. The author mentions twelve principles which can be used by medical educators to create COVID-19 friendly learning design. Some of the ideas addressed were known to me some of the others are new. I am of the opinion that educators can benefit from a practical demonstration of some of these ideas and software may be in the form of a webinar. These are important concepts some of which are not well known to medical and other educators. The SPARKPlus peer review system to gauge student contributions to their group seems promising. I am also interested in learning more about the online application called Vocaroo which can help in delivering online feedback to students. There are some other software and applications which seem to be useful. This is an important paper and is richly supported by references from the literature.

Reviewer Expertise:

NA

No decision status is available

# Reviewer report: Reviewer response for version 1

**DOI:** 10.21956/mep.20071.r30839

**Published:** 2020-05-20

**Authors:** John Sandars

**Affiliations:** 1 Edge Hill University

This review has been migrated. The reviewer awarded 4 stars out of 5 This is a timely article that highlights the importance of learning design for the effective use of technology to enhance teaching and learning. The tips are practical and supported by relevant references. The article could have usefully discussed the importance of iterative /agile design, with rapid cycles that focus on both the usefulness and usability of the approach being developed.Overall, a quick reminder of how to design online learning.

Reviewer Expertise:

NA

No decision status is available

# Reviewer report: Reviewer response for version 1

**DOI:** 10.21956/mep.20071.r30836

**Published:** 2020-05-20

**Authors:** Megan Anakin

**Affiliations:** 1 University of Otago

This review has been migrated. The reviewer awarded 4 stars out of 5 Thank you for writing this useful article that we can share with our clinical teaching staff and our medical school and with colleagues in other health professional programmes. The tips are well-grounded in the education literature and in the educational technology and medical education literature in particular. As we emerge from 'lockdown' in New Zealand, we too are wondering how we might sustain (and possibly enhance) the gains made to our programme during our urgent transition to online learning.

Reviewer Expertise:

NA

No decision status is available

# Reviewer report: Reviewer response for version 1

**DOI:** 10.21956/mep.20071.r30837

**Published:** 2020-05-20

**Authors:** Stuart Marshall

**Affiliations:** 1 Monash University

This review has been migrated. The reviewer awarded 4 stars out of 5 This is a great summary of what to consider in our socially distanced post COVID educational world.Would be great to include more tips around using existing social media resources such as blogs, podcasts and #FOAMed resources on Twitter etc.Encouraging to see reference to assessment strategies too. Are 'take home' and 'open book' exams too controversial for medicine?!

Reviewer Expertise:

NA

No decision status is available
